# Commentary: The energy model of insulin resistance: a unifying theory linking seed oils to metabolic disease and cancer

**DOI:** 10.3389/fnut.2025.1622160

**Published:** 2025-08-05

**Authors:** Miguel López-Moreno

**Affiliations:** Diet, Planetary Health and Performance, Faculty of Health Sciences, Universidad Francisco de Vitoria, Madrid, Spain

**Keywords:** seed oil, insulin resistance, cancer, inflammation, metabolic disease, omega-6 fatty acid

The article “*The energy model of insulin resistance: A unifying theory linking seed oils to metabolic disease and cancer*” by Shanahan (1) presents a provocative hypothesis regarding the role of refined, bleached, and deodorized (RBD) seed oils in the etiology of insulin resistance and related metabolic diseases. This hypothesis was previously outlined in her book *Dark Calories: How Vegetable Oils Destroy Our Health and How We Can Get It Back*, in which she even compares the effects of seed oils to smoking cigarettes. The proposed model places oxidative stress at the center of chronic metabolic dysfunction, suggesting that modern levels of dietary polyunsaturated fatty acids (PUFA), particularly from RBD oils, may exceed what our physiology can tolerate and thereby promote insulin resistance, pre-diabetes, and other chronic conditions. While this perspective challenges conventional dietary assumptions and offers a mechanistic link between PUFA intake and rising disease prevalence, several critical methodological and conceptual deficiencies undermine the scientific rigor and impact of the manuscript.

## 1 Lack of causal evidence and methodological rigor in support of the article's claims

The article's central thesis—that RBD seed oils are a primary driver of insulin resistance and cancer—is not supported by direct experimental or clinical evidence. The arguments rely heavily on epidemiological correlations and historical trends in dietary fat consumption, without providing controlled intervention studies or mechanistic experiments that demonstrate causality in humans. Figure 1 in Shanahan (1) lacks scientific rigor as it implies a causal relationship based solely on temporal association without statistical contrast—a logical fallacy known as *post hoc ergo propter hoc* (2). This fallacy assumes that if one event follows another, the first must be the cause of the second, without accounting for confounding variables or alternative explanations. Furthermore, the use of disparate units (e.g., pounds/year, ounces/day, and percent prevalence) on the same graph is misleading and prevents meaningful quantitative comparisons. Concluding this type of visualization and arguments risks oversimplifying public health issues and misinforming audiences about evidence-based dietary determinants of chronic disease.

## 2 Dismissal of evidence-based guidelines and the broader scientific consensus

The author critiques the American Heart Association (AHA) and the Dietary Guidelines for Americans regarding their fat intake recommendations. However, the manuscript fails to offer a balanced critique or recognize the complex and ever-evolving nature of nutritional science. While the arguments presented in this study question the saturated fat–cholesterol hypothesis, emphasizing only studies that report no association between saturated fat intake and cardiovascular disease risk may reflect selective interpretation. The overall scientific evidence, as consistently demonstrated in comprehensive analyses such as the meta-analysis conducted by institutions such as Cochrane and the World Health Organization, supports reducing saturated fat intake and replacing it with unsaturated fats to lower cardiovascular risk (3, 4). The dismissal of decades of research and expert consensus without a thorough and systematic counter-analysis is a significant shortcoming.

## 3 Energy model of insulin: failure to acknowledge contradictory evidence and overstatement of seed oil risks

The article's central argument is built upon the Energy Model of Insulin Resistance, which posits a mechanistic relationship between dietary inputs and cellular energy metabolism. Specifically, the model suggests that the intake of RBD seed oils promotes cellular oxidative stress, thereby compelling cells to adapt their fueling strategy to mitigate oxidative damage. The manuscript does not provide any human studies to support the claim that “replacing animal fats with RBD seed oils promotes cellular oxidative stress,” which underpins the author's hypothesis. This statement lacks nuance and may overstate the available evidence. A recent prospective population-based cohort study with 221,054 adults and 33 years of follow-up observed that the substitution of butter with vegetable oils, such as soybean oil, is associated with a lower risk of all-cause mortality (5). Similarly, a recently published systematic review of clinical studies analyzed the effects of seed oils on metabolic health, including glycemic control and inflammatory markers (6). This systematic review found that seed oils have a positive impact on fasting blood glucose levels, insulin sensitivity, and GLUT-4 gene expression. Additionally, the review highlighted the potential role of these oils in modulating oxidative stress markers, contributing to improved inflammation profiles. These findings contradict the central argument of Shanahan (1), which asserts that seed oils promote cellular oxidative stress, thereby disrupting glucose homeostasis and leading to insulin resistance. Based on the available scientific literature, the American Diabetes Association (ADA), in its *Standards of Care in Diabetes*−*2025*, recommends cooking with vegetable oils (e.g., canola and olive oil) in place of fats high in saturated fat (e.g., butter, shortening, lard, and coconut oil) (7). Taken together, the current body of scientific evidence does not support the hypothesis that seed oils have deleterious effects on metabolic health in humans and, in fact, suggests potential benefits when used as a replacement for saturated fats.

## 4 Speculative claims linking PUFA intake to cancer

The article also alludes to a possible link between PUFA and cancer, referencing early observations by Efraim Racker regarding mitochondrial uncoupling and hypothetical toxic effects of long-term PUFA intake. While historical perspectives such as Racker's (13) editorial may be of anecdotal interest, they do not constitute robust evidence, nor do they justify causal claims about cancer risk. Furthermore, the article cites “evidence from the largest and most well-controlled randomized human clinical trial” suggesting that RBD seed oil increased cancer and overall mortality. This appears to refer to a re-analysis of the Minnesota Coronary Experiment (8); however, that study did not evaluate cancer outcomes nor directly assess the relationship between RBD seed oils and cancer. Importantly, current evidence from prospective cohort studies does not support an increased risk of cancer with higher n-6 PUFA intake; on the contrary, higher blood levels of these fatty acids have been associated with a lower risk of developing cancer (9).

## 5 Unsubstantiated carbohydrate-insulin model: lack of evidence for causality in diabetes pathophysiology

The hypothesis underlying the manuscript, which relies on the carbohydrate-insulin model (CIM), has been extensively tested in prior research without being substantiated. Multiple randomized controlled trials, including short-term interventions in inpatients with high internal validity (10), as well as longer trials such as the DIETFITS study (11), have failed to demonstrate a causal relationship between carbohydrate intake and impaired glycemic control or increased energy intake. Additionally, a recent study testing the CIM assessed short-term metabolic responses to meals with varying glycemic index (GI) in healthy adults (12). The findings revealed no significant differences in subjective hunger among the different GI groups and no effect of GI on subsequent meal intake.

Taken together, these results undermine the central premise of the carbohydrate-insulin model and do not support its application as an explanatory framework for glucose dysregulation or diabetes pathophysiology.

## 6 Discussion

In light of the available scientific literature, public health recommendations must be grounded in a comprehensive understanding of the current evidence base. The body of evidence consistently supports the substitution of saturated fats with unsaturated fats, such as those derived from seed oils, as a strategy to reduce the risk of prevalent and serious health conditions like cardiovascular disease and diabetes. While individual hypotheses and theories may provide insight into specific aspects of metabolic health, it is important to recognize that the broader scientific consensus, including guidelines from esteemed organizations like the ADA and the AHA, reflects the accumulated knowledge from diverse fields of study. In this context, the growing popularity of books that promote alarmist and scientifically unsubstantiated claims—such as equating the consumption of seed oils with smoking cigarettes—represents a concerning trend.
